# Association of HCG Level with Ultrasound Visualization of the Gestational Sac in Early Viable Pregnancies

**DOI:** 10.1007/s43032-023-01308-7

**Published:** 2023-08-10

**Authors:** Kristen E. Park, Kyle R. Latack, Nicole L. Vestal, Sue A. Ingles, Richard J. Paulson, Michael S. Awadalla

**Affiliations:** 1 Keck School of Medicine, University of Southern California, Los Angeles, CA USA; 2 Department of Obstetrics and Gynecology, University of Michigan, Ann Arbor, MI USA; 3 Department of Pediatrics, Children’s Hospital of Los Angeles, Los Angeles, CA USA; 4 Department of Population and Public Health Sciences, Keck School of Medicine, University of Southern California, Los Angeles, CA USA; 5 Division of Reproductive Endocrinology and Infertility, Department of Obstetrics and Gynecology, Keck School of Medicine, University of Southern California, Los Angeles, CA USA; 6 Institute for Reproductive Health, 3805 Edwards Rd Suite 450, Cincinnati, OH 45209 USA

**Keywords:** HCG, Early pregnancy, Gestational sac, Yolk sac, Discriminatory zone

## Abstract

Our primary objective is to verify or refute a 2013 study by Connolly et al. which showed that in early pregnancy, a gestational sac was visualized 99% of the time on transvaginal ultrasound when the HCG level reached 3510 mIU/mL. Our secondary objective was to make clinical correlations by assessing the relationship between human chorionic gonadotropin (HCG) level in early pregnancy when a gestational sac is not seen and pregnancy outcomes of live birth, spontaneous abortion, and ectopic pregnancy. This retrospective study includes 144 pregnancies with an outcome of live birth, 87 pregnancies with an outcome of spontaneous abortion, and 59 ectopic pregnancies. Logistic regression is used to determine the probability of visualizing a gestational sac and/or yolk sac based on the HCG level. A gestational sac is predicted to be visualized 50% of the time at an HCG level of 979 mIU/mL, 90% at 2421 mIU/mL, and 99% of the time at 3994 mIU/mL. A yolk sac was predicted to be visualized 50% of the time at an HCG level of 4626 mIU/mL, 90% at 12,892 mIU/mL, and 99% at 39,454 mIU/mL. A total of 90% of ectopic pregnancies presented with an HCG level below 3994 mIU/mL. These results are in agreement with the study by Connolly et al. Since most early ectopic pregnancies had an HCG value below the discriminatory level for gestational sac visualization, other methods for the evaluation of pregnancy of unknown location such as repeat HCG values are clinically important.

## Introduction

Understanding the relationship between early pregnancy, gestational sac development, and human chorionic gonadotropin (HCG) rise is essential for managing an early pregnancy and differentiating between normal pregnancy, spontaneous abortion, and ectopic pregnancy. The concept of an HCG discriminatory cutoff above which a gestational sac should be seen on ultrasound was first mentioned in 1981 by Kadar et al. and was based on transabdominal ultrasound imaging [1]. In 2013, Connolly et al. published the most commonly referenced modern study on discriminatory levels of serum HCG in early pregnancy assessed by transvaginal ultrasound (TVUS) [2]. They studied women who presented to an emergency department between 2007 and 2009 with pain or bleeding in early pregnancy and went on to have a viable pregnancy. They concluded that the gestational sac can be seen 1% of the time on TVUS when the HCG is 390 mIU/mL and 99% of the time when the HCG is 3510 mIU/mL. The discriminatory value of 3510 mIU/mL was higher than those found by previous studies and suggested a transition zone where, with increasing HCG, the likelihood of visualizing the gestational sac increased [3–7]. For this reason, when a pregnancy of unknown location is diagnosed (no gestational sac seen in the uterus on transvaginal ultrasound), the risk of ectopic pregnancy increases with increasing HCG values. Despite being nearly a decade old, this data appears to have never been verified.

The Connolly et al. study analysis has several limitations that limit its utility in subsequent research and clinical practice. First, there is no discussion of the assessment of the logit assumption or the evaluation of the goodness of fit of the logistic regression model. Second, the discussion of fractional polynomials (which found HCG^0.5^ to be the best-fit model for gestational sac and the linear model to be the best fit for yolk sac) is very limited. Third, their study only included viable pregnancies and did not evaluate HCG levels at presentation for patients with no gestational sac on ultrasound who went on to have spontaneous abortions or ectopic pregnancies. Lastly, the raw data is not available which limits the ability of others to combine data from multiple studies to form larger datasets for analysis.

Our primary objective was to independently verify or refute the findings of Connolly et al. regarding the HCG values and the probability of visualizing a gestational sac or yolk sac in early viable pregnancies. Our secondary objective was to make clinical correlations by assessing the relationship between HCG in early pregnancy when a gestational sac is not seen and the outcomes of live birth, spontaneous abortion, and ectopic pregnancy.

## Methods

### Study Population

Patients were identified retrospectively through labor and delivery and ob/gyn triage records at Los Angeles General Medical Center (formerly Los Angeles County Medical Center) for patients presenting from May 2016 to December 2020. This county hospital uses a single medical record system in the outpatient, inpatient, and emergency room settings. A viable pregnancy was defined as either a live birth, a normal pregnancy at greater than 20 weeks gestational age, or fetal heart tones documented at follow-up. A spontaneous abortion was defined as a confirmed abortion or no fetal heart tones documented at follow-up. Finally, ectopic pregnancy was defined as either a confirmed or presumed ectopic pregnancy. At this center, suction dilation and curettage is routinely performed to rule out intrauterine pregnancy prior to medical treatment of ectopic pregnancy.

**Fig. 1:**
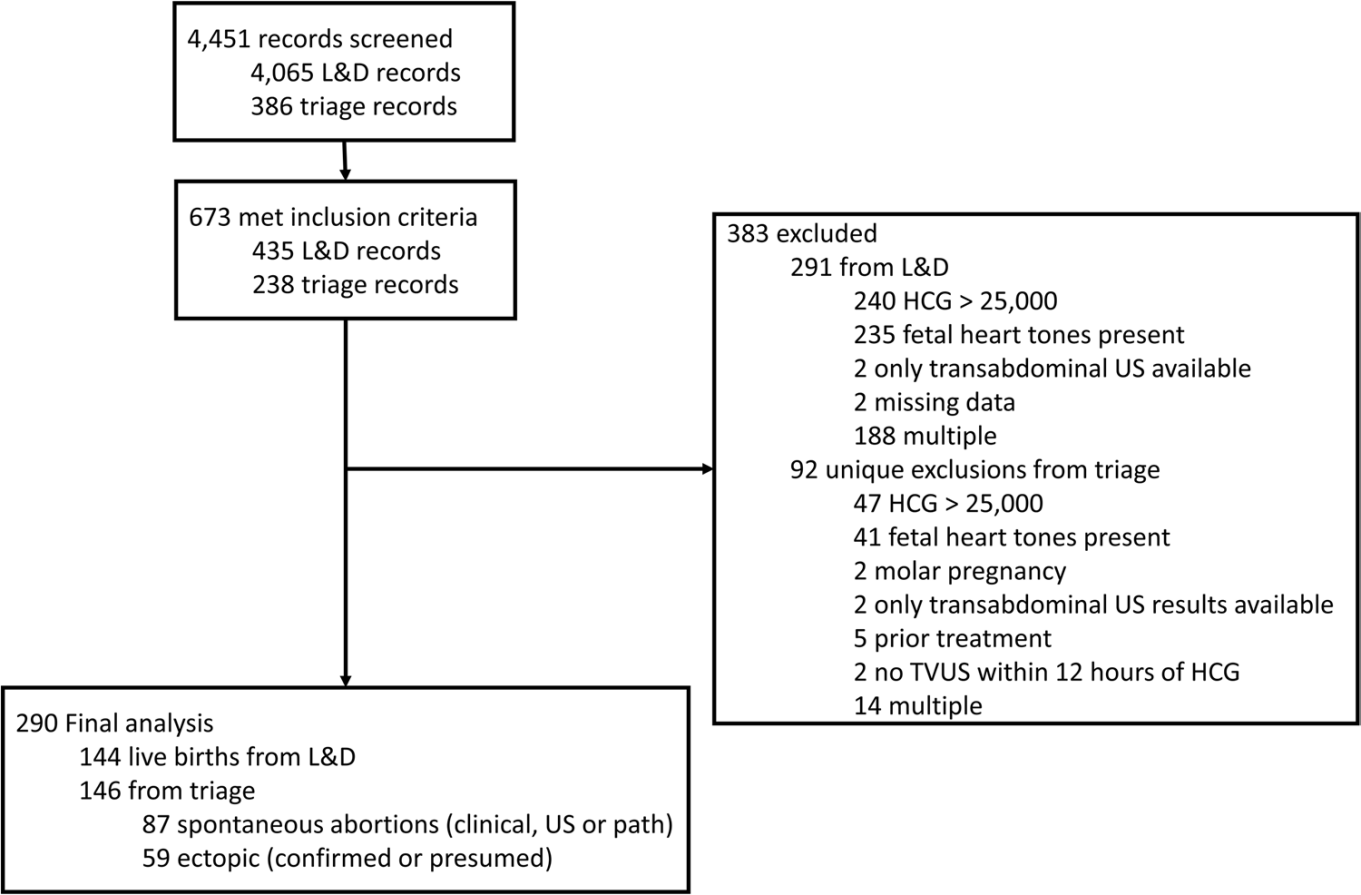
Flow diagram with inclusion and exclusion criteria

Patients meeting inclusion criteria were between the ages of 12 and 55 years old and must have had either a TVUS and HCG test within 12 h of each other or a TVUS between two HCG tests less than 72 h apart. For patients with a TVUS performed between two HCG tests, the HCG at the time of the TVUS was determined by linear interpolation. Exclusion criteria included molar pregnancies, unknown pregnancy outcomes, multiple gestations, only transabdominal ultrasound results, uterus visualization obstructed by large fibroids, prior medical or surgical abortion treatment earlier in the pregnancy, HCG greater than 25,000, and fetal heart tones documented on TVUS. A total of 290 patients met the inclusion criteria, as shown in Fig. 1. The mean age was 30.0 years (SD 7.1 years), and the mean BMI was 29.8 kg/m^2^ (SD 7.0 kg/m^2^). The study population was 72% Hispanic, 8% African American, 4% Asian, 1% Caucasian, 8% other, and 7% unknown. A total of 89% of the transvaginal ultrasounds were performed by a sonographer in the radiology department, 10% by a supervised ob/gyn resident, 1% by a fellow, and 1% by an attending.

HCG was measured using the Roche Diagnostics cobas e 801 analyzer using the Elecsys HCG+β assay. This assay has intraassay and interassay coefficients of variation of under 5%. Serum HCG values are reported standardized against the 4th International Standard for Chorionic Gonadotropin from the National Institute for Biological Standards and Control (NIBSC) code 75/589. This assay measures the sum of HCG plus the HCG β-subunit in serum or plasma including the whole hormone, nicked HCG, the β-core fragment, and the free β-subunit.

Of patients who presented more than once, only data from the initial ultrasound was used in the study. The initial data included was reviewed for outliers, and approximately 15% of the initial data was determined to be possible outliers. These charts were reviewed again to verify if the inclusion criteria were met. This study was approved by the University of Southern California IRB (HS-19-00829).

### Statistical Analysis

For pregnancies resulting in a live birth, logistic regression was used to model the probability that a gestational sac or yolk sac would be visualized as a function of HCG level (Stata version 16.1, StataCorp, College Station, TX). We used fractional polynomials to determine how best to model the relationship between HCG and visualization of the gestational sac and yolk sac. We evaluated the linearity assumption (the assumption of a linear relationship between HCG and the logit) by constructing LOWESS plots. This was done separately for visualization of the gestational sac and for visualization of the yolk sac.

The linear model was the best model for visualization of the gestational sac. The linearity assumption was valid only when HCG was less than 5000 mIU/mL, and we therefore restricted our analysis to this range. Connolly et al. used HCG^0.5^ in their model. While we did find that HCG^0.5^ was the best fit 1° model for the prediction of the gestational sac, it was not significantly better than the linear model (*p* = 0.23). For this reason, we used the simpler linear model shown below for predicting the probability (*p*) of visualizing the gestational sac.

$$\ln \frac{p}{1-p}=a+b\ast \textrm{HCG}$$

The natural logarithm transformation of HCG provided the best-fit model for visualization of the yolk sac. The natural logarithm of HCG was linearly related to the logit for all HCG values up to 25,000 mIU/mL. Connolly et al. used the linear model for predicting the probability of visualization of the yolk sac. We found that ln(HCG) was significantly better than the linear model (*p* < 0.01). For this reason, we use the natural logarithm of HCG in the model for predicting the probability (*p*) of visualizing the yolk sac.

$$\ln \frac{p}{1-p}=a+b\ast \ln \left(\textrm{HCG}\right)$$

In these equations, *p* is the probability of seeing a gestational sac or yolk sac, *a* is the constant term, and *b* is the coefficient of HCG or ln(HCG). The Hosmer and Lemeshow overall goodness of fit tests did not show evidence of poor fit for predicting the presence of a gestational sac (*p* = 0.39) or a yolk sac (*p* = 0.48) [8]. We are providing access to all of the data and detailed statistical methods used to perform and evaluate the logistic regression modeling in Stata through Mendeley Data [9].

## Results

Out of 4451 records reviewed, 290 met all criteria for the study, as shown in Fig. 1. Of the included pregnancies, 144 resulted in live birth, 87 in spontaneous abortion, and 59 in ectopic pregnancy. Twenty of the spontaneous abortions presented as pregnancies of unknown location with no visible gestational sac on the initial transvaginal ultrasound.

**Fig. 2:**
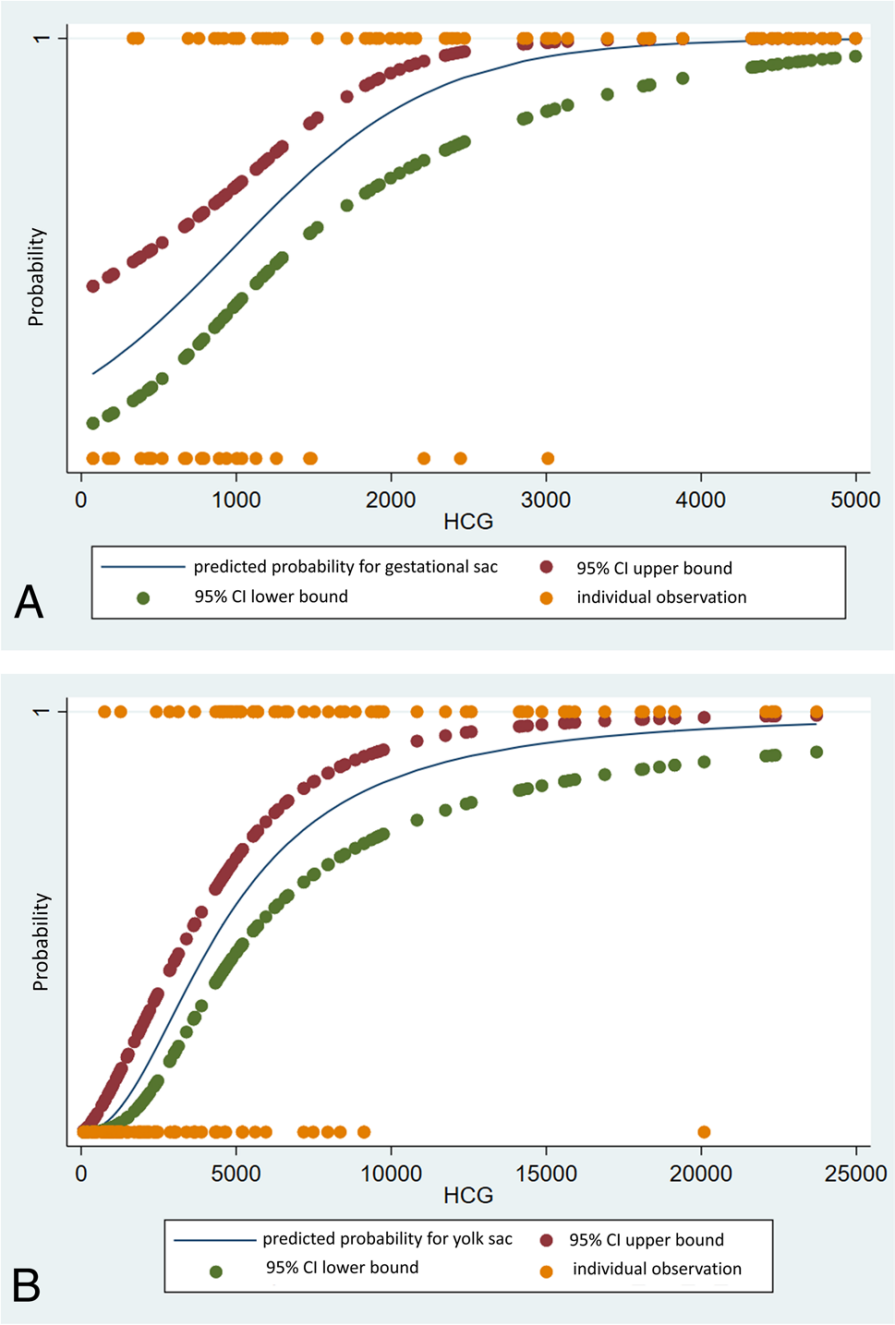
Predicted probability of detecting a gestational sac (**A**) and yolk sac (**B**) based on HCG in early viable pregnancy

Of pregnancies that resulted in live births, a gestational sac was predicted to be visualized 50% of the time at an HCG level of 979 mIU/mL, 90% at 2421 mIU/mL, 95% at 2911, and 99% of the time at 3994 mIU/mL. A yolk sac was predicted to be visualized 50% of the time at an HCG level of 4626 mIU/mL, 90% at 12,892 mIU/mL, 95% at 18,268, and 99% at 39,454 mIU/mL (Fig. 2). These values are shown in comparison to those reported by Connolly et al. in Table 1, and a graphical representation of their corresponding logistic regressions is shown in Fig. 2.

**Table 1:** Comparison of serum HCG levels and predicted probability of detection of gestational sac and yolk sac for Connolly et al. (*n* = 366) and the current study at Los Angeles General Medical Center (*n* = 144)

	50%	90%	95%	99%
Gestational Sac Visualization				
LA General	979	2421	2911	3994
Connolly et al.	879	1918	2363	3510
Yolk Sac Visualization				
LA General	4626	12,892	18,268	39,454
Connolly et al.	1826	5412	7832	17,716

The best-fit logistic regression models are shown below.

Pr﻿obability (*p*) of visualization of gestational sac:

$$\ln \frac{p}{1-p}=-1.492463+0.0015243\ast \textrm{HCG}$$

Probability (*p*) of visualization of yolk sac:

$$\ln \frac{p}{1-p}=-18.09216+2.143768\ast \ln \left(\textrm{HCG}\right)$$

**Fig. 3:**
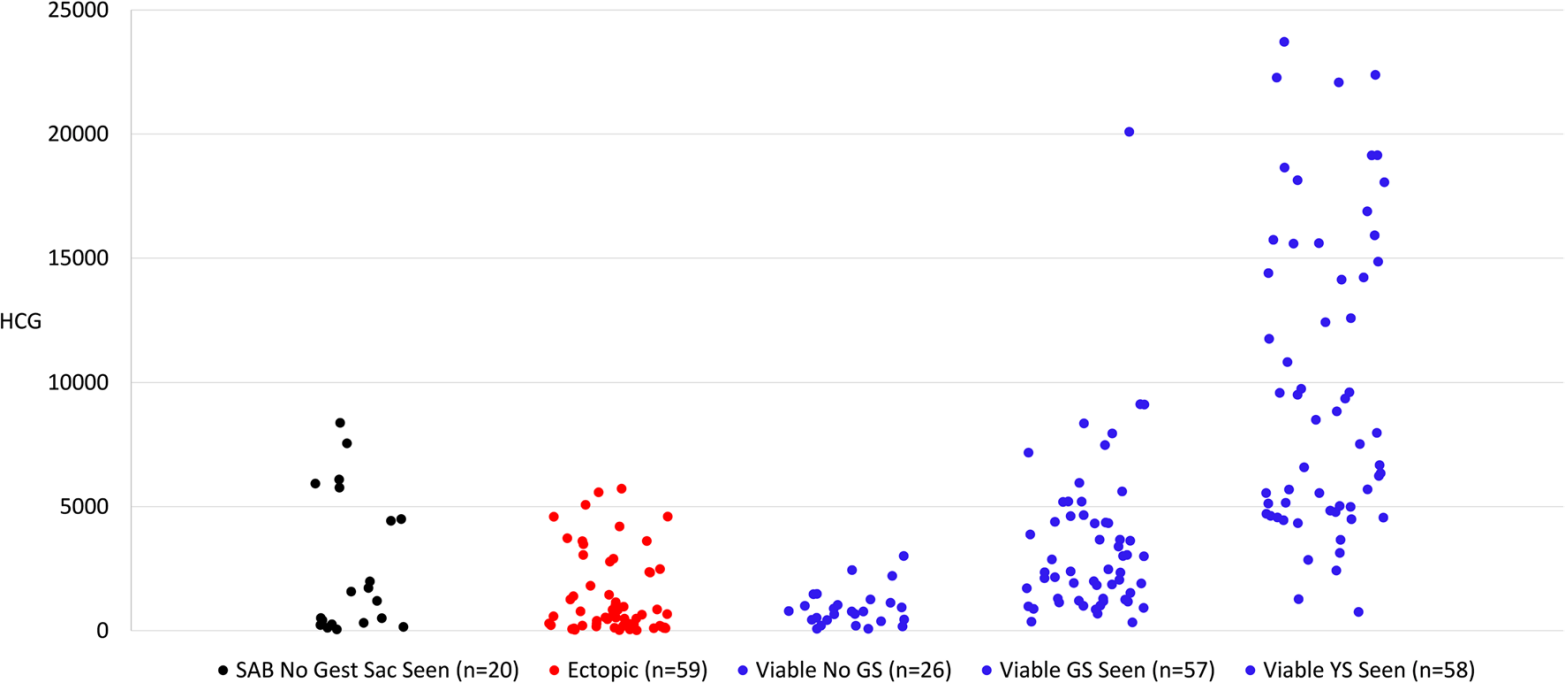
Comparison of HCG values and early pregnancy findings at presentation categorized by eventual pregnancy outcome. The first 3 groups on the left would be considered pregnancies of unknown location. 67 spontaneous abortions had gestational sacs and are not included. Three viable pregnancies had a yolk sac and crown-rump length without fetal heart tones and are not included. SAB, spontaneous abortion; GS, gestational sac; YS, yolk sac

HCG values for early pregnancies and their corresponding outcomes are shown in Fig. 3. In Fig. 3, the three groups on the left would all be considered to be a pregnancy of unknown location [10]. Only 6 of 59 (10%) ectopic pregnancies had a HCG above the 99% threshold for detection of the gestational sac of 3994 mIU/mL. Presenting symptoms for the pregnancies of unknown location in Fig. 3 are shown in Table 2.

**Table 2:** Presenting signs of pregnancy of unknown location and pregnancy outcome

	Pain only	Bleeding only	Both	Neither
SAB (*n*= 20)	3 (15%)	4 (20%)	10 (50%)	3 (15%)
Ectopic (*n* = 59)	10 (17%)	7 (12%)	35 (59%)	7 (12%)
Viable (*n* = 26)	13 (50%)	0	5 (19%)	8 (31%)

## Discussion

The HCG values at which the gestational sac is predicted to be seen based on this study are similar to those reported by Connolly et al., with the discriminatory level for visualization of the gestational sac being slightly higher (3994 mIU/mL here compared to 3510 mIU/mL). The highest reported HCG value with no gestational sac seen on transvaginal ultrasound is 9083 mIU/mL for a patient with a triplet pregnancy and 4336 mIU/mL for a singleton pregnancy [11, 12].

The HCG values for visualizing a yolk sac in this study are higher than those found in the Connolly study. The HCG level for predicting visualization of a yolk sac 99% of the time of 39,454 mIU/mL is within the confidence interval found in the Connolly et al. study. Even though the Connolly et al. study has a larger sample size, the confidence interval reported in their study is still wide since the sample size is relatively small. One of the biggest limitations of this study is the sample size. Our sample size was limited as we were not able to review data from before 2016.

Even in the modern era of medicine where electronic medical records are the norm, compiling data on pregnancies with known outcomes and corresponding ultrasound and HCG values in early pregnancy is a laborious task. The initial study by Kadar et al. in 1981 examined the records of 53 patients. The Connolly et al. study had a sample size of 366 patients who presented with pain or bleeding and went on to have a viable pregnancy. In this study, we examined records of patients who had a live delivery and retrospectively collected data on those with recorded early TVUS and HCG. We also reviewed the gynecology triage records of patients presenting for evaluation of early pregnancies to obtain data on early ectopic pregnancies and spontaneous abortion. This allowed for in-depth clinical correlation which can help evaluate pregnancies of unknown location.

Figure 3 includes the HCG values for pregnancies with no gestational sac on initial TVUS that went on to become a spontaneous abortion. It is likely that some of these pregnancies have high HCG values with no intrauterine gestational sac because the gestational sac had already passed. These could be clinically confused with ectopic pregnancy because some of these pregnancies presented with HCG above the 99% discriminatory level of 3994 mIU/mL. Serial HCG measurement is helpful for detecting spontaneous abortion with a high presenting HCG level because the level typically decreases at a mean rate of 70–75% over 2 days [13].

As seen in Fig. 3, most of the ectopic pregnancies presented with an HCG value under 2000 mIU/mL. We found that 90% of ectopic pregnancies had an HCG at presentation that was less than the value at which 99% of viable pregnancies can be detected by visualizing a gestational sac on transvaginal ultrasound (3994 mIU/mL in this study). Few of the ectopic pregnancies included would have qualified for immediate intervention (medical or surgical treatment for ectopic pregnancy) based on HCG above the discriminatory level at the time of presentation. Table 2 shows that there is a significant symptom overlap between pregnancies of unknown location regardless of the eventual pregnancy outcome (SAB, ectopic, or viable pregnancy). More emphasis should be placed on repeat HCG values for early detection of ectopic pregnancy in addition to assessing clinical presentation.

The commonly accepted practice of assessing pregnancy of unknown location by repeating the HCG after 2 days dates back to a 1981 study by Kadar et al. [14]. The 48-h sampling interval was recommended because “after 1 day, the difference between the mean percent hCG increase of intrauterine and ectopic pregnancies (20%) is less than twice the interassay variability.” The interassay and intraassay coefficients of variation cited in the 1981 Kadar et al. study is “less than 15%.” Since modern-day assays have coefficients of variation of 5% or less, repeating the HCG after 24 h is appropriate in modern practice. This would allow for more rapid management and could decrease the risk of a ruptured ectopic pregnancy.

This study is accompanied by the dataset used for the logistic regression as well as all of the Stata code to completely recreate the statistical analysis [9]. This allows other investigators to use our methods with their datasets or to combine datasets from multiple studies.

In conclusion, this study is in agreement with the 2013 study by Connolly et al. since the logistic regression model for this data predicts that 99% of early viable singleton pregnancies will have a visible gestational sac on transvaginal ultrasound when the HCG level reaches 3994 mIU/mL. This limits the utility of a discriminatory level to detect ectopic pregnancies. Since only 10% of ectopic pregnancies included in this study had an HCG value above 3994 mIU/mL, the discriminatory level concept is not very useful in detecting ectopic pregnancies in modern practice. We feel that rapid repeat HCG measurement (such as repeating after only 24 h) is an underutilized strategy for evaluating early pregnancies and is appropriate based on modern HCG assays that are much more precise than they were in the 1980s.

### Availability of Data and Material

Limited data used to perform the logistic regression is provided through Mendeley Data.

### Code Availability

The Stata code used for statistical analysis is provided through Mendeley Data.
